# In-Situ Self-Encapsulated Tin-Halide Perovskites for Air-Functional Near-Infrared Light-Emitting Diodes

**DOI:** 10.1021/acsenergylett.5c01017

**Published:** 2025-06-23

**Authors:** Heyong Wang, Antonella Treglia, Chun-Sheng Jack Wu, Guanhaojie Zheng, Miguel M. de Vries Ibáñez, Gianvito Vilé, Hui Li, Luca Gregori, Filippo De Angelis, Jianpu Wang, Feng Gao, Annamaria Petrozza

**Affiliations:** † Center for Nano Science and Technology, 403543 Istituto Italiano di Tecnologia, via Rubattino 81, Milano 20134, Italy; ‡ Shanghai Synchrotron Radiation Facility (SSRF) Zhangjiang Lab, Shanghai Advanced Research Institute, Chinese Academy of Sciences, Shanghai 201204, China; § Department of Chemistry, Materials, and Chemical Engineering “Giulio Natta”, 18981 Politecnico di Milano, Piazza Leonardo da Vinci 32, IT-20133 Milano, Italy; ∥ Department of Chemistry, Biology and Biotechnology, 9309 University of Perugia, Via Elce di Sotto 8, Perugia 06123, Italy; ⊥ Key Laboratory of Flexible Electronics (KLOFE), Institute of Advanced Materials (IAM) & School of Flexible Electronics (Future Technologies), 91599 Nanjing Tech University (NanjingTech), Nanjing 211816, China; # Department of Physics, Chemistry and Biology (IFM), 4566 Linköping University, 58183 Linköping, Sweden; □ Computational Laboratory for Hybrid/Organic Photovoltaics (CLHYO), Istituto CNR di Scienze e Tecnologie Chimiche “Giulio Natta” (CNR-SCITEC), Perugia 06123, Italy; ○ SKKU Institute of Energy Science and Technology (SIEST), Sungkyunkwan University, Suwon 440-746, South Korea

## Abstract

Tin-halide perovskites are emerging as exceptional materials for near-infrared light-emitting diodes (NIR-LEDs). However, their extreme oxygen sensitivity remains a significant obstacle to practical applications. This work presents a facile yet effective strategy to overcome this limitation by designing self-encapsulated tin-halide perovskite films. Incorporating a rational molecule, 4,4′-diaminodiphenyl sulfone, into precursors, it forms isolated tin-iodide perovskite particles that are encapsulated in situ, achieving outstanding air stability. The resulting films show high crystallinity, reduced trap density, and mitigated p-doping density, boosting radiative charge recombination to reach an impressive photoluminescence quantum yield approaching 50%. Leveraging these advancements, the resulting NIR-LEDs demonstrate a record-breaking peak external quantum efficiency of 12.4%, accompanied by a substantial improvement in operational lifetime. Notably, for the first time, we demonstrated a functional tin-iodide perovskite-based device in ambient air. This work provides a robust pathway for realizing high-performance and stable tin-halide perovskite-based optoelectronic devices, addressing critical challenges for their widespread application.

## Graphical abstract

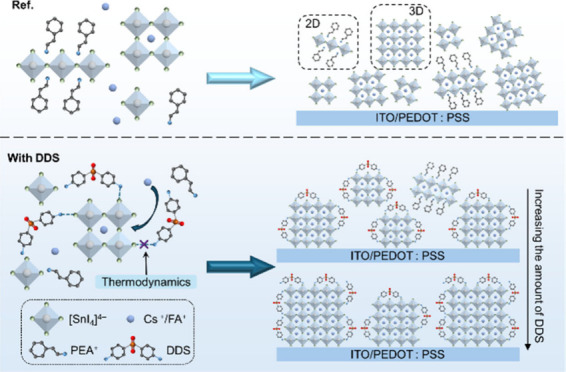

Near-infrared light-emitting diodes (NIR-LEDs) have garnered significant interest for diverse applications including night vision, biomedical treatments, optical communication, and data storage. Commercial NIR-LEDs predominantly utilize epitaxial heterostructures of III–V inorganic semiconductors as emitters. Despite achieving nearly 100% internal quantum efficiency, they face limitations stemming from complex material processing and device architectures, one over all, the difficulty of integration in multicomponent photonic and electronic systems.^1–3^ Recent advancements in NIR-LEDs employing solution-processed and vacuum-deposited materials, such as organic semiconductors and colloidal quantum dots, have offered a higher level of resilience for integration.^4^ However, the development of NIR-LEDs in the NIR-I and NIR-II ranges remains modest due to fundamental bandgap constraints of light-emitting materials.^5–11^

Metal-halide perovskites have emerged as outstanding light-emitting materials due to their highly tunable bandgaps, simple and versatile synthesis, and exceptional optoelectronic properties. With extensive efforts, while lead-halide perovskites have demonstrated remarkable efficiency and excellent color purity, their emission wavelengths are inherently capped at ∼800 nm.^12^ Tin-halide perovskites offer a significant advantage by extending emission wavelengths to ∼1000 nm,^13,14^ effectively covering the biological transparency window (650–950 nm) and the telecom window (800–900 nm). Recent advancements in low-dimensional structures,^15^ solvent engineering,^16,17^ and additives engineering^18–20^ have improved the efficiency of tin-halide perovskite-based NIR-LEDs. However, the instability of tin-halide perovskites continues to present a major challenge, impeding their effective exploitation in optoelectronic devices.

The instability of tin-halide perovskites is primarily driven by the easy oxidation of Sn cations, which enrich the thin film surface of electron trapping defects (Sn^4+^) and uncontrollably p-dopes the semiconductor. This leads to carrier loss through trap-mediated and Auger nonradiative recombination processes.^21,22^ To mitigate this issue, various strategies have been developed to manage precursors and growth process, including increasing oxidation potential (e.g., utilizing large-sized A cations, mixed Pb^2+^ and Sn^2+^ cations),^23–25^ loading reductants (e.g., metallic Sn, SnF_2_, and SnCl_2_),^26^ passivating surface defects.^27–29^ Nevertheless, tin-halide perovskite thin films still undergo rapid oxidation and degradation upon exposure to trace amounts of air, posing a persistent challenge for practical applications.^30^

This work presents an effective strategy that leads to a self-encapsulated perovskite thin film with enhanced stability and optoelectronic quality. By incorporating a rationally designed organic molecule (4,4′-diaminodiphenyl sulfone, DDS) into the perovskite precursors, we induce the in situ formation of self-encapsulated perovskite films, effectively suppressing Sn^2+^ cation oxidation even under ambient conditions. This approach not only significantly improves air stability but also reduces trap carrier density and allows for control of the p-doping densities in the semiconducting films, yielding a remarkable photoluminescence quantum yield (PLQY) of nearly 50%. Leveraging the high quality of these thin films, we fabricated NIR-LEDs with a record-breaking peak external quantum efficiency (EQE) of 12.4% and extended operational lifetimes with half-lifetime (T_50_: the time it takes until the light output decreases to 50% of the maximum output) over 1 h at a constant current density of 10 mA cm^–2^. Notably, for the first time, this device demonstrates functionality in ambient air without external encapsulation. These achievements establish a new benchmark for tin-halide perovskite-based optoelectronic devices, showcasing the potential of molecular design to address intrinsic perovskite materials challenges. Our findings open new pathways for the practical deployment of tin-halide perovskites in high-performance and stable optoelectronic applications, marking a significant step forward in next-generation NIR technologies.

## Stability of Tin-Iodide Perovskite Thin Films

First, we investigated the stability of tin-iodide perovskite thin films by monitoring their photoluminescence (PL) spectra in air without external encapsulation. As shown in Figure 1, the tin-iodide perovskite thin film without reductants or additives degrades almost immediately, with a negligible PL signal observed. The tin-iodide perovskite thin film incorporating SnF_2_ and metallic Sn shows extended PL emission for only a few minutes. Incorporating additional PEAI, a commonly used additive to enhance the performance of tin-halide perovskite-based LEDs and solar cells,^18,31,32^ slightly extends the PL emission to 10 min but it is accompanied by the formation of 2D tin-iodide perovskite phases. Remarkably, the tin-iodide perovskite thin film incorporating DDS retains 60% of its initial PL intensity after 100 min, with a monotonous decrease over 10 h. These findings demonstrate that DDS plays a dominant role in improving the air stability of tin-iodide perovskite thin films, representing a critical step toward the realization of stable, high-performance tin-halide perovskite-based optoelectronic devices.

**Figure 1:**
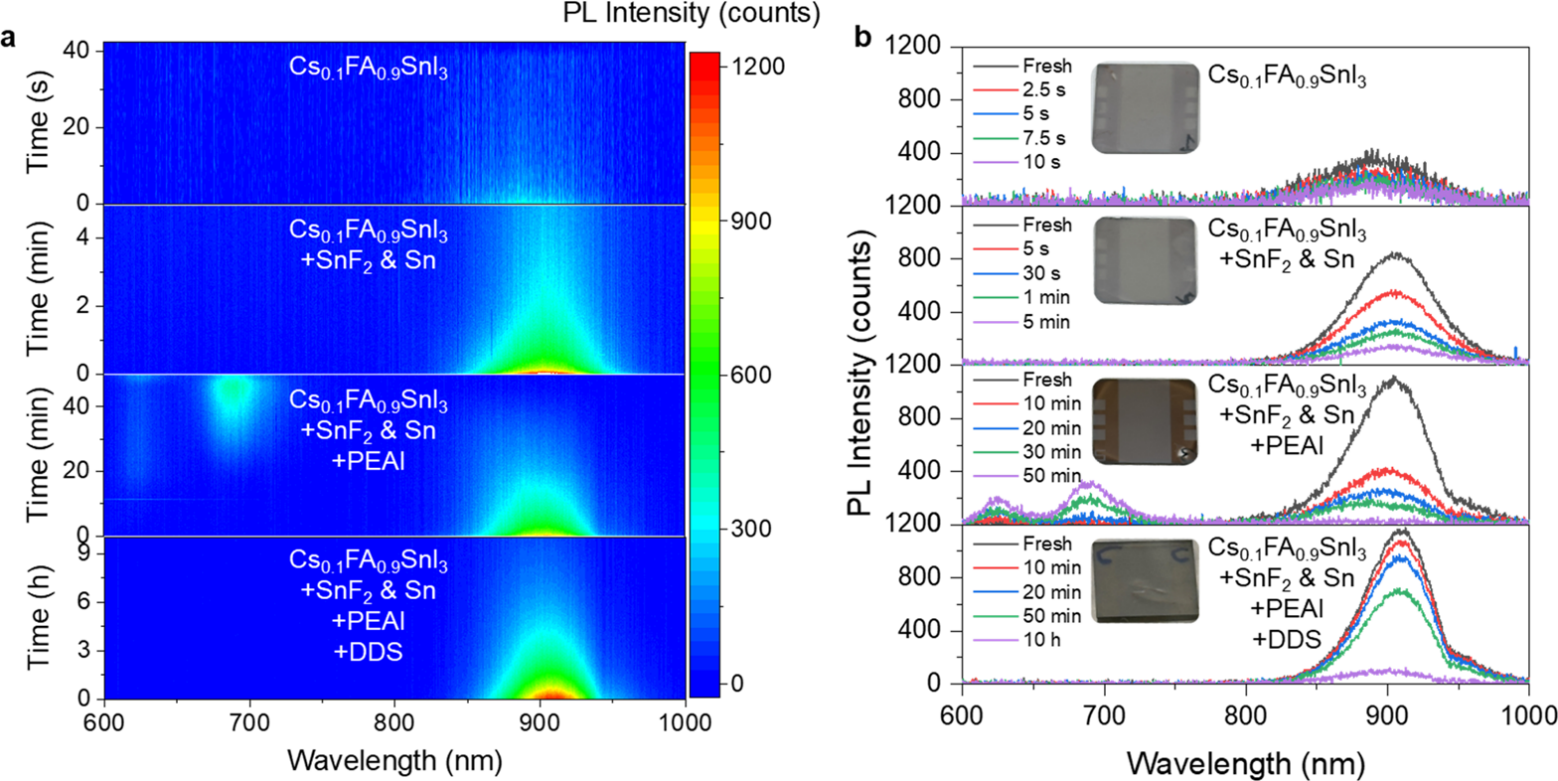
Stability of perovskite films in ambient air. **a** PL mappings and **b** spectra at different time of perovskite films in time since they exposed to air.

## Thin Film Characterization

To understand the reasons behind the improved air stability, we characterized the tin-iodide perovskite thin film. First, Scanning Electron Microscopy (SEM) is performed to visualize the morphology of optimized tin-iodide perovskite film (the ratio between DDS and SnI_2_ is 1:1). As shown in Figure 2a, the stable tin-iodide perovskite thin film (deposited from precursors containing the building blocks of perovskites, SnF_2_, metallic Sn, PEAI, and DDS), hereafter named as “With DDS”, show isolated bright particles, which look like “Lucky 4-Leaf Clover”. We confirmed the composition of the thin film with energy-dispersive X-ray spectroscopy (EDS) by analyzing the signals of iodine­(I) (characteristic elements of tin-iodide perovskite) and sulfur (S) (characteristic element of DDS) spectra. The line-scan EDS along the black line shows that the normalized intensities of the I elements sharply increase at the edge of the bright particle and remain constant throughout the particle, indicating that the bright particle is a tin-iodide perovskite. In contrast, the intensity of the S element decreases on top of bright particles. Additionally, we performed high-resolution EDS by averaging the signal across three regions (as shown in Figure S2): outside the particle (R1 – blue line), on the particle (R2 – red line), and across the entire line scan (including both outside and on the particles – black line). Figure 2b shows the zoom-in spectra of S element from a selected range of the stable tin-iodide perovskite thin film with DDS. The weak intensity of S from R2 implies a very thin DDS layer on top of perovskites.

**Figure 2:**
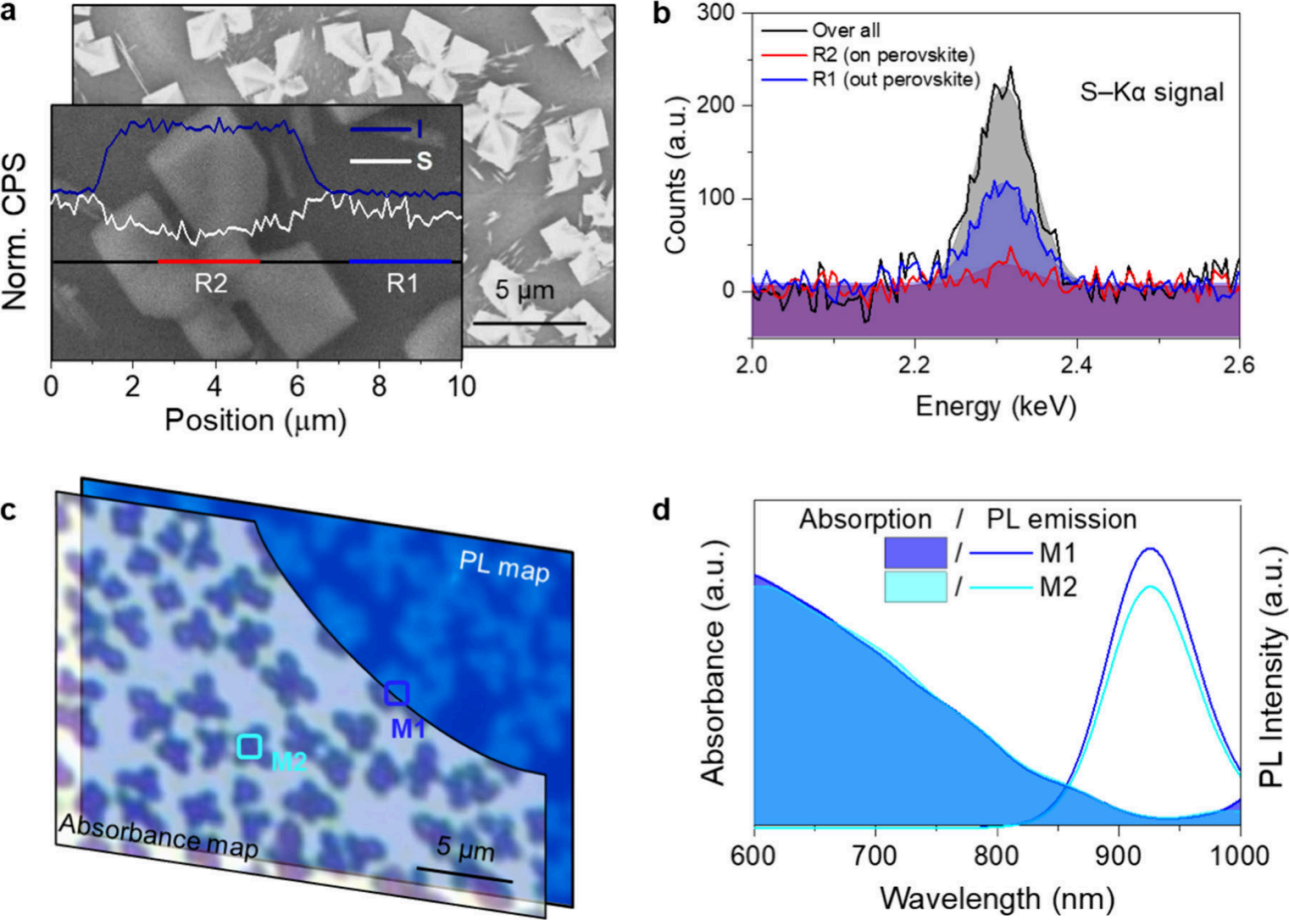
Characterization of the air-stable tin-iodide perovskite thin film. **a** Top-view SEM image of the stable tin-iodide perovskite thin film. (Inset: Normalized intensity of I and S elements along the line scan.) **b** The zoomed-in spectra of S element from a selected range of the stable tin-iodide perovskite thin film with DDS. **c** Absorption and PL (emission center at 910 nm) maps and **d** the spectra extracted from selected ranges of the stable tin-iodide perovskite thin film.

Furthermore, we performed absorbance and PL mapping on the tin-iodide perovskite thin films with DDS to assess their optical properties. As shown in Figure 2c, the isolated perovskite particles correspond to spatial regions with a higher absorbance and photoluminescence. The absorbance and PL spectra extracted from randomly representative particles (M1 and M2, marked in Figure 2c) reveal the characteristic absorption and emission profiles of 3D tin-iodide perovskites (Figure 2d). The nearly identical absorption and PL profiles of perovskites (slightly different intensities fall within the experimental error range) illustrate the uniformity of perovskite particles across the film. These results let us conclude that high-quality isolated tin-iodide perovskite particles are embedded within the DDS matrix, forming a self-encapsulated structure, which would lead to significantly enhanced air stability.

## NIR-LED Performances

We fabricated NIR-LEDs based on the hybrid composite thin film, with the following device architecture:^33^ indium tin oxide (ITO)/poly­(3,4-ethylenedioxythiophene):poly­(styrenesulfonate) (PEDOT:PSS)/emitting layer/2,2′,2′-(1,3,5-benzinetriyl)­tris­(1-phenyl-1*H*-benzimidazole) (TPBi)/lithium fluoride (LiF)/aluminum (Al) as shown in Figure 3a. The current density–voltage-radiance characteristics of the device are shown in Figure 3b, which presents low current density, ranging from 1.5 × 10^–5^ to 1.2 × 10^–4^ mA cm^–2^, in the ohmic region (from 0 to 1.7 V). Compared to the device without DDS (Figure S3a), the optimized NIR-LED with DDS shows significantly reduced leakage current even with isolated tin-iodide perovskite particles, which can be attributed to the DDS refilled space between perovskite particles. The ultrathin DDS layer (which is an electron-transport molecule with a high singlet energy (2.95 eV), triplet energy (3.59 eV),^34^ HOMO (−5.16 eV), and LUMO (−1.36 eV) as shown in Figure S4), on top of perovskites allows for efficient carrier injection (carrier tunneling dominates the carrier transport), resulting in a sharp onset from 1.8 V (injection-limited region) and detected electroluminescence (EL) spectrum from 2.0 V (recombination region). A maximum radiance of 24 W m^–2^ sr^–1^ is achieved at 5 V (166 mA cm^–2^). The EL spectra (peaking at 909 nm with fwhm of 61 nm) show negligible shift under various driving voltages (Figure 3c) demonstrating good EL spectral stability.

The NIR-LEDs based on a self-encapsulated tin-iodide perovskite thin film demonstrate both high efficiency and prolonged operational stability. As shown in Figure 3d and Figure S5a, the peak EQE significantly improves with incremental additions of DDS, reaching a maximum value of 12.4% at an optimized DDS:SnI_2_ molar ratio of 1:1 in the precursors solution. Notably, the device retains a high EQE of 8.7% (70% of the peak value) even under elevated current density operation at a high current density of 156 mA cm^–2^, demonstrating robust performance under high-bias conditions and low efficiency roll-off. The peak EQEs histogram of the optimized device is shown in Figure S5b, demonstrating good reproducibility of the fabricated devices. The optimized NIR-LED shows an operational stability (Figure 3e) with a *T*_50_ of over 1h at a constant current density of 10 mA cm^–2^ (the current density over that of reaching peak EQE), which is much longer than that of the NIR-LEDs based on tin-iodide perovskite film without DDS (Figure S3d). This device shows the highest efficiency reported to date, and it retains it for a long time under operation (Figure 3f and Table S1). Notably, for the first time, the tin-iodide perovskite-based devices demonstrate functionality in ambient air (*T*_50_: 1.5 min at a constant current density of 10 mA cm^–2^) without external encapsulation (Figure S5c), albeit for limited durations.

**Figure 3:**
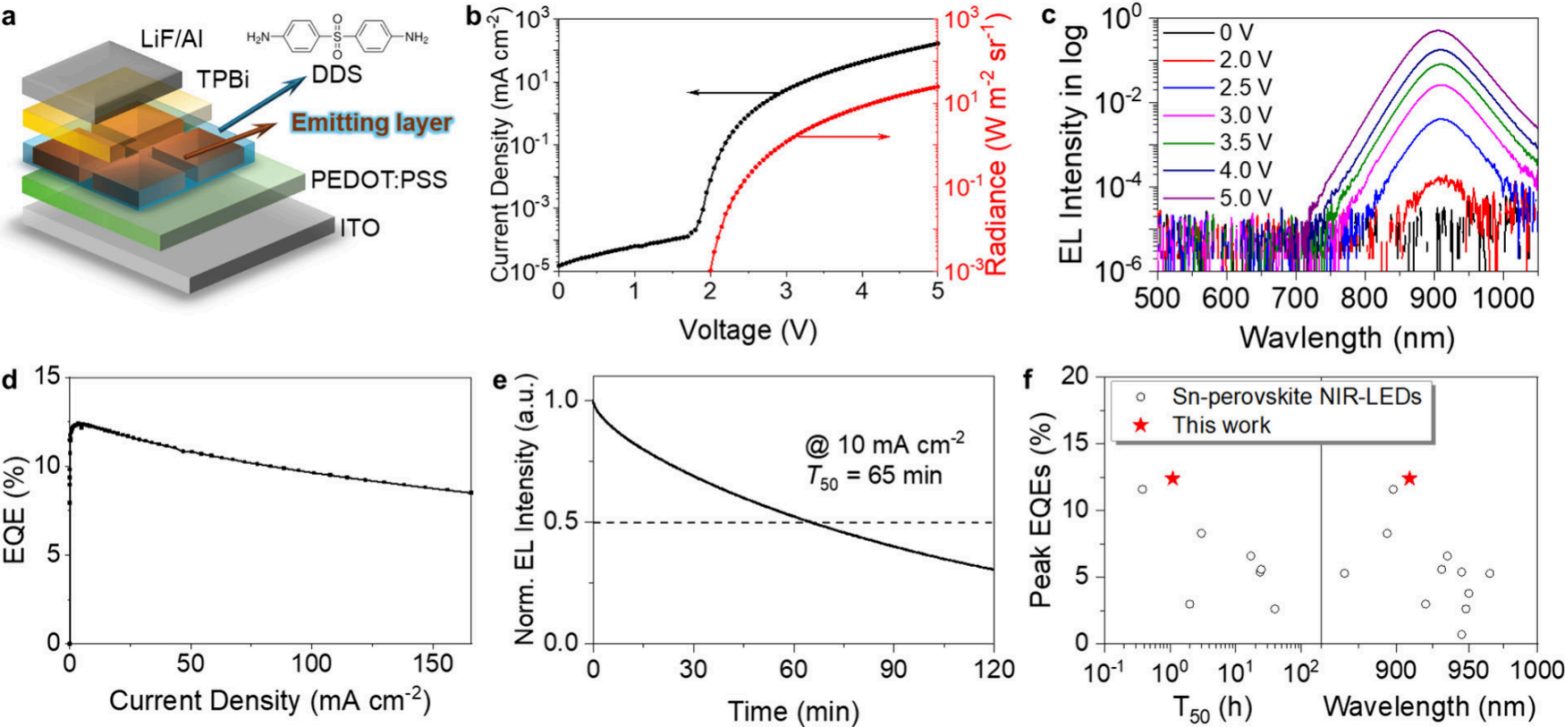
Device performance of NIR-LEDs based on the stable tin-halide perovskites measured in a N_2_-filled glovebox. **a** Schematic illustration of the NIR-LEDs structure. **b** Current density–voltage-radiance curves, **c** EL spectra at various voltage, **d** EQE–current density curve, and **e** operational stability of the optimized NIR-LEDs based on the stable tin-iodide perovskite thin film. **f** Reported peak EQEs as a function of half-lifetime and EL peaks wavelength of NIR-LEDs based on tin-halide perovskites as provided in Table S1.

## Role of Chemical Interactions within the Hybrid Matrix to Enhance Stability

The remarkable results obtained motivated us to investigate the role of DDS, offering insights into future molecular design. Unlike widely studied long-chain alkyl additives in lead- and tin-halide perovskites, which typically passivate surface traps by chemically bonding with FA^+^ cations,^18,35–37^ DDS, a rigid aniline derivative, chemically bond with I^–^ anions. This is confirmed by attenuated total reflectance-Fourier transform infrared (ATR-FT-IR) spectroscopy (Figure 4a), which shows no change in the stretching vibration (ν­(C–N)) of FA^+^ before and after the incorporation of DDS. Instead, as shown in Figure S6a, the electron-withdrawing benzene and sulfonyl groups in DDS reduce the electronegativity of the −NH_2_ groups, enabling them to interact with I^–^ anions. Evidence for this interaction is provided by a shift of the scissoring vibration of −NH_2_ (δ­(NH_2_)­DDS) to a lower wavenumber after binding with perovskites, indicating weakened N–H bonds. First-principles calculations further reveal the adsorption configurations of DDS on tin-iodide perovskites.^38–41^ The most stable configuration is achieved when DDS binds to the SnI_2_-terminated surface through two −NH_2_ groups, with hydrogen atoms pointing toward I^–^ anions (Figure 4b). This configuration enhances structural stability, as confirmed by adsorption energy calculations (Table S2). To explore the influence of DDS’s electron-withdrawing groups, we replaced them with weaker fluorene groups (Figure S6b), using 2,7-diaminofluorene (DAF). As shown in Figure S6c,d, while the resulting films are continuous and show improved NIR-LED EQEs, they degrade immediately upon exposure to air. These findings highlight the critical importance of rationally tuned chemical bonding energies between additives and perovskites for achieving self-encapsulated thin films with enhanced stability and performance.

## In-Situ Formation of the Self-Encapsulated Tin-Iodide Perovskite Thin Films

Next, we investigated the formation mechanism of the hybrid composite. The color changes of perovskite films during spin-coating and thermal annealing provide intuitive information about the crystallization time. As shown in Figure 4c, the perovskite film deposited from precursors without DDS (hereafter named Ref.) rapidly changes from colorless to light brown within 40 s during the spin-coating process. Thermal annealing process does not alter the color of the reference perovskite film. In contrast, the perovskite films with DDS remain colorless after 1 min of spin-coating. The perovskite films with +1.0 DDS begin to change to gray after 10 s of thermal annealing, a delay that increases with higher DDS concentrations—16 s for +2.0 DDS (where +1.0 DDS and +2.0 DDS denote the molar ratio of DDS to SnI_2_ in precursors). To gain more insights, we conducted in situ PL measurements during the spin-coating process. As shown in the integrated PL spectra (Figure S7), the Ref. film exhibited PL emission 30 s after spin-coating commenced, suggesting that tin-iodide perovskites formation began during the process, which correlates with the observed color change. In contrast, the +1.0 DDS perovskite film showed no PL emission during spin-coating, with emission appearing only 10 s after the process ceased. The +2.0 DDS films showed a further delay in the PL appearance. These findings clearly indicate that DDS significantly retards the crystallization and growth of tin-iodide perovskite thin films.

Then we characterized the deposited tin-iodide perovskite thin films with varying amounts of DDS. As shown in Figure S8, the Ref. film without DDS shows random bright blocks and irregular dark flats in SEM image, indicating poor morphology. In contrast, the optimized film for NIR-LEDs containing DDS shows well-defined perovskite particles as discussed above. As the DDS content in the precursors increases, the perovskite particles grow anisotropically, forming elongated “Clover”-like structures with longer “leaves.” Furthermore, the crystallization behavior of these films was analyzed using grazing-incidence wide-angle X-ray scattering (GIWAXS). As shown in Figure 4d, the film exhibits weak and uniform diffraction intensity along the azimuth angle at *q* = 10 nm^–1^, corresponding to the (100) plane of 3D tin-iodide perovskites. In contrast, perovskite films containing DDS display distinct diffraction mottling at the dominant diffraction ring of *q*_*z*_ = 10 nm^–1^, indicating long-range-orientated crystallization of the 3D perovskite phase along the out-of-plane direction. Integrated GIWAXS (Figure S9) reveals strong diffraction peaks at low *q* values (2.7 and 5.2 nm^–1^) in the Ref. film, corresponding to 2D tin-iodide perovskites likely caused by excess PEAI.^35^ Notably, with +1.0 DDS, the peak at 2.7 nm^–1^ disappears, and at higher DDS concentrations both peaks vanish entirely. These results demonstrate that DDS promotes the formation of a highly crystalline 3D perovskite phase with preferred orientation along the out-of-plane direction.

**Figure 4:**
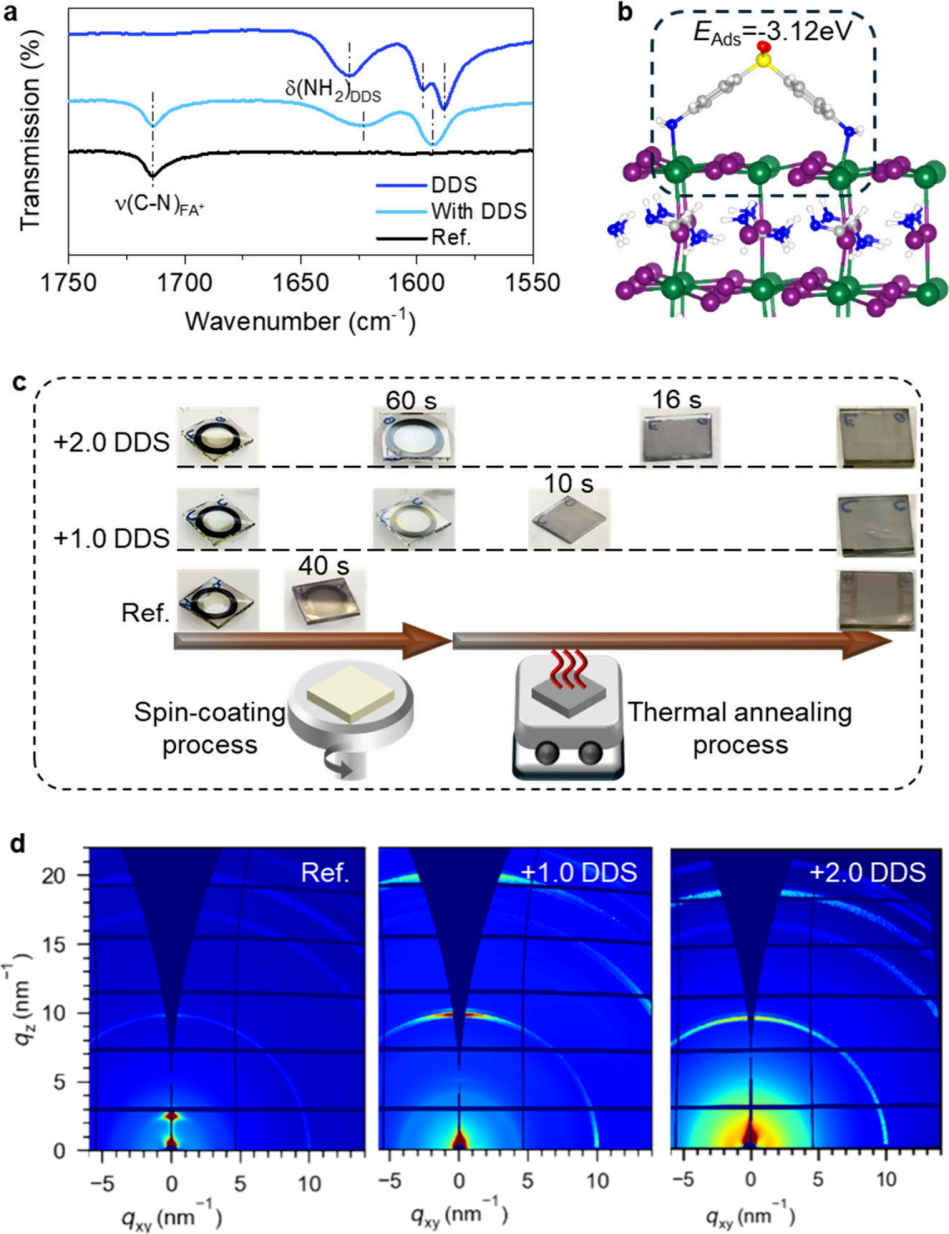
Chemical interactions. **a** FTIR spectra of DDS, tin-iodide perovskite films without (ref 1) and with DDS. **b** Adsorption configuration of DDS on tin-iodide perovskites. **c** Optical pictures of the tin-iodide perovskite films without (ref) and with varying amounts of DDS during spin-coating and thermal annealing process. **d** GIWAXS patterns of tin-iodide perovskite films without (ref) and with varying amounts of DDS.

Hence, we indicate that DDS retards the growth of the tin-iodide perovskite film. In Ref. films, rapid crystallization leads to random growth of 2D and 3D perovskite phases. In contrast, DDS retards the crystallization process by chemically binding to surface [SnI_2_] sites. During thermal annealing, the weak hydrogen bonds between DDS and the perovskite break, allowing the FA^+^/Cs^+^ cations to incorporate and form the 3D perovskite phase. Excess DDS remains on the perovskite particle surfaces and fills the interparticle gaps, creating a self-encapsulated structure that enhances film stability and performance.

## Carrier Dynamics of Tin-Iodide Perovskite Films

The carrier dynamics of tin-iodide perovskite thin films were investigated to evaluate their quality as light-emitting materials. In both the Ref. film and the film incorporating DDS, carrier recombination is predominantly governed by the 3D perovskite phase, resulting in single PL emission spectra, as shown in Figure S10. The absolute PLQY as a function of excitation density, presented in Figure 5a, shows a significant improvement for the perovskite film with DDS, reaching a peak value of 45% (compared with 19% for the reference film). Moreover, the PLQY peak of the perovskite film with DDS is shifted to lower carrier density (∼10^17^ cm^–3^ compared to ∼10^18^ cm^–3^ for the reference film), qualitatively indicating a lower density of deep electronic states within the bandgap. The PL dynamics, measured at high excitation densities, just before the PLQY starts dropping because of multiparticle interactions, move from few to tens of nanoseconds when the DDS is added (Figure 5b) unambiguously demonstrating the reduced Auger recombination in the self-encapsulated perovskite film with DDS.

**Figure 5:**
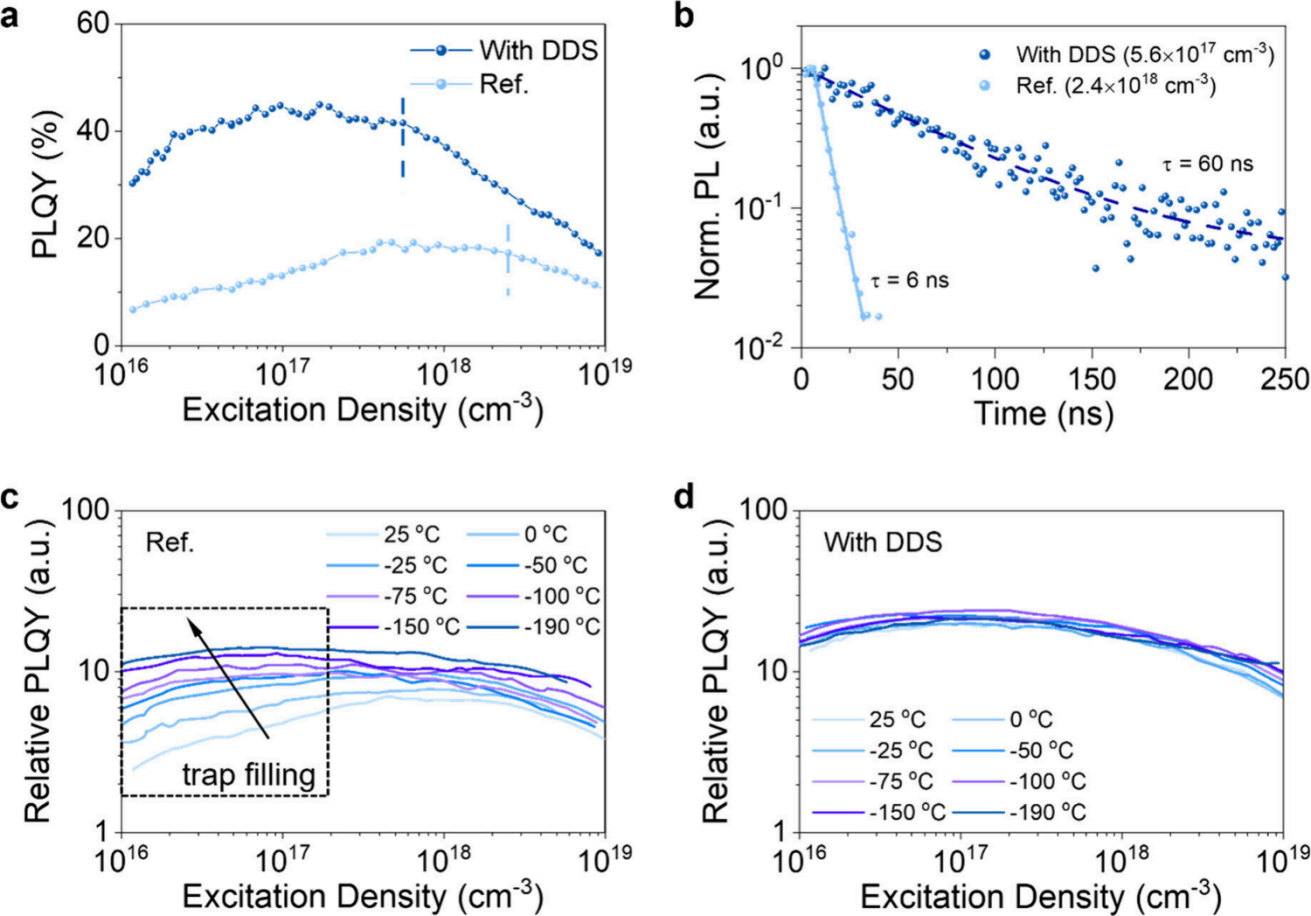
Carrier dynamics of the perovskite films. **a** Absolute PLQYs and **b** normalized PL intensity decay of tin-iodide perovskite films without (Ref.) and with DDS. Relative PLQY curves as a function of excitation density at various temperatures of tin-iodide perovskite films **c** without (Ref.) and **d** with DDS.

To further investigate and assess the overall impact of carrier trap states and p-type doping on carrier dynamics, we employed transient absorption (TA) spectroscopy. Figure S11 presents the TA spectra and the free carrier dynamics obtained by integrating the photobleach (PB) signal from the TA spectra. In brief, the PB band is assigned to free carriers’ band filling and is proportional to the density of electrons and holes populating, upon photoexcitation, the bottom/top of the conduction/valence band, respectively. The self-encapsulated perovskite film exhibits a single dynamic. It follows the one obtained from the time-resolved PL experiments and becomes shorter and shorter as the excitation density increases. This suggests that we are following the band-to-band bimolecular carrier recombination.^42^ Notably, the reference sample shows a dynamic at early times that follows the PL decay, with little dependence on the excitation density, and a very long tail in μs time window, which is a dark dynamic. They are a signature of an electronics doping mediated recombination process (fast dynamics)^22,42^ and a trap mediated recombination process (long-lived one).^30^ Such an observation clearly indicates a high electronic quality of the self-encapsulated tin-iodide perovskites, where both the uncontrolled self-p-doping and the trap density are reduced. To quantify these quantities, we simulated the TA decays and PLQY curves using a kinetic model that includes both p-doping and deep carrier traps.^42^ The simulation results, shown in Figure S12, reveal a trap density of 5 × 10^15^ cm^–3^ and a p-doping density of 2 × 10^18^ cm^–3^ for the self-encapsulated tin-iodide perovskite thin film with DDS, compared to 6 × 10^16^ and 1 × 10^19^ cm^–3^ for the reference perovskite film.

We performed temperature-dependent PLQY measurements to provide additional insights into the carrier recombination processes in the films. In Figure 5c,d, we show the relative PLQYs of the reference sample and the self-encapsulated tin-iodide perovskite thin film with DDS, respectively, as a function of the excitation density, in a temperature range which goes from +25 to −190 °C. Notably, at room temperature, in the low photoexcitation regime, i.e., below 10^18^ cm^3^, the reference sample shows a steeper slope of the PL intensity signal, which increases almost by a factor of 10 when it reaches its peak value, thus revealing a clear trap mediated recombination process. In agreement, as the temperature is reduced, the absolute value of the PL signal increases, and the curve becomes almost flat. In fact, the free photogenerated carriers do not have enough thermal energy to be trapped in localized energy states. On the other hand, the self-encapsulated tin-iodide perovskite thin film with DDS, which shows a PLQY peak doubling the one of the reference film, at room temperature already shows an almost flat curve of the PL intensity vs excitation density and it shows a very little change over temperature, thus demonstrating minimal role of trap mediated recombination. It is important to highlight that, though the self-doping level of the semiconductor is reduced when the perovskites crystallize in the presence of DDS, the semiconductor remains p-type doped. The term describing radiative recombination in the rate equation of photocarrier recombination, *k* × *n*(*t*) × *p*(*t*) (where *p*(*t*)/*n*(*t*) is the total instantaneous population of free holes/electrons) includes two processes: the bimolecular recombination of photoexcited electrons and holes and the pseudo-monomolecular radiative recombination of photogenerated electrons with dopant holes. The latter has a positive effect on the radiative efficiency of the material. Thus, the capability of tuning the doping and trap density, in order to be in such regime to boost the radiative recombination, is the key to improve efficiency in doped-perovskite LEDs.

In summary, we demonstrated a facile yet effective strategy for depositing an air-stable tin-iodide perovskite thin film, resulting in both improved efficiency and stability of NIR-LEDs. By introduction of rationally designed organic molecules into perovskite precursors, the crystallization process of tin-iodide perovskites is substantially retarded, promoting the formation of highly crystallized 3D tin-iodide perovskites along the out-of-plane direction and resulting in low traps density and p-doping density. Meanwhile, DDS molecules fully encapsulate the tin-iodide perovskites, protecting them from air exposure and improving their phase stability. Though further experimental evidence is needed, given the very low thickness of the DDS layer, we believe that it does not work as a simple barrier which does not allow water and oxygen to permeate but it chemically interacts with the perovskite surface and slow down the Sn oxidation process. Then, the ultrathin DDS layer on top of tin-iodide perovskite particles has minimal impact on carrier injection when employing the thin film as emitting layer in NIR-LEDs, enabling a high peak EQE of 12.4% and a half-lifetime exceeding 1 h. Notably, this device demonstrates functionality in ambient air without external encapsulation. This work demonstrates the potential of integrating perovskites with a wide variety of molecular strategies to address the persistent challenges of tin-halide perovskites and paves the way for future advancements in optoelectronic devices.

## Supplementary Material



## Notes

Experimental details, materials, and methods; material characterizations such as PL, EDS, EL, excitation and emission, and TA spectra, current density–voltage–radiance curves, CV, EQEs, SEM, integrated GIWAXS spectra; device performances, adsorption energy difference (PDF)
