# Supplementary material for: In-Situ Self-Encapsulated Tin-Halide Perovskites for Air-Functional Near-Infrared Light-Emitting Diodes
Source: ACS Energy Lett. 2025 Jun 23;10(7):3375–82. doi: 10.1021/acsenergylett.5c01017 (PMC12261328; doi:10.1021/acsenergylett.5c01017)
Supplement: Supplementary file 1 [file nz5c01017_si_001.pdf]

Supporting Information

In-Situ Self-Encapsulated Tin-Halide Perovskites for Air-Functional Near-Infrared Light-Emitting Diodes

*Heyong Wang[1], Antonella Treglia[1], Chun-Sheng Jack Wu[1], Guanhaojie Zheng[2], Miguel M. de Vries Ibáñez[3], Gianvito Vilé[3], Hui Li[1], Luca Gregori[4,7], Filippo De Angelis[4,7,8], Jianpu Wang[5], Feng Gao[6], Annamaria Petrozza[1]**

[1] Center for Nano Science and Technology, Istituto Italiano di Tecnologia, via Rubattino 81, Milano, 20134, Italy

[2] Shanghai Synchrotron Radiation Facility (SSRF) Zhangjiang Lab, Shanghai Advanced Research Institute, Chinese Academy of Sciences, Shanghai, 201204, China

[3] Department of Chemistry, Materials, and Chemical Engineering "Giulio Natta", Politecnico di Milano, Piazza Leonardo da Vinci 32, IT-20133 Milano, Italy

[4] Department of Chemistry, Biology and Biotechnology, University of Perugia, Perugia, 06123, Italy

[5] Key Laboratory of Flexible Electronics (KLOFE), Institute of Advanced Materials (IAM) & School of Flexible Electronics (Future Technologies), Nanjing Tech University (NanjingTech), Nanjing, 211816, China

[6] Department of Physics, Chemistry and Biology (IFM), Linköping University, 58183, Linköping, Sweden

[7]Computational Laboratory for Hybrid/Organic Photovoltaics (CLHYO), Istituto CNR di Scienze e Tecnologie Chimiche "Giulio Natta" (CNR-SCITEC), Perugia, 06123, Italy;

[8]SKKU Institute of Energy Science and Technology (SIEST), Sungkyunkwan University, Suwon, 440-746, South Korea;

Corresponding Author

Annamaria Petrozza− Center for Nano Science and Technology, Istituto Italiano di Tecnologia, 20134 Milano, Italy; orcid.org/0000-0001-6914-4537;

Email: annamaria.petrozza@iit.it

Methods

Material. Chemicals listed below are commercially available and used without further purification. Tin(II) iodide (SnI_2, 99.999%), tin(II) fluoride (SnF_2, 99%), 4,4′-Diaminodiphenyl sulfone, Lithium fluoride, TPBi, dimethylformamide (DMF), and dimethyl sulfoxide (DMSO) were purchased from Sigma-Aldrich. Formamidinium iodide (FAI), Caesium iodide (CsI, 99.999%), and phenethylamine iodide (PEAI) were purchased from TCI. PEDOT:PSS (AI 4083) was purchased from Ossila.

Perovskite precursors. The perovskite precursors were prepared by dissolving FAI, CsI, PEAI, SnI_2, and DDS with a molar ratio of 0.9 : 0.1 : 0.3 : 1 : x in a mixed solvent DMF/DMSO (v/v = 4 : 1). x = 0 and 1 are corresponding to tin-iodide perovskite film without and with DDS, respectively. The concentration of perovskite precursors is defined by the concentration of SnI_2 (0.3M). Extra SnF_2 (0.06M) and metallic Sn (0.02M) were used as reductants. The perovskite precursors were put on a hot plate and stirred at room temperature overnight, followed by filtering the solution with 0.22 μm PTFE filter.

Perovskite film and device preparation. ITO-coated glasses were cleaned by sonication in deionized water, acetone, and 2-Propanol for 15 min each, then dried under a nitrogen flow. Ultraviolet ozone is used to treat the surface of ITO for 10 min. The PEDOT:PSS (diluted by deionized water with a v/v=1:9) hole injection layer was spin coated on ITO at 5,000 r.p.m for 30 s, followed by annealing in air at 150 °C for 10 min. Transferring the substrate into a nitrogen-filled glovebox. The tin-iodide perovskite films were deposited by spin-coating the tin-iodide perovskite precursors on the substrate at 5000 r.p.m for 60 s, followed by annealing at 80 °C for 5 min. The NIR-LEDs were fabricated by sequentially depositing TPBi (40 nm), LiF (1 nm), and Al (100 nm) through thermal evaporation.

Device characterizations. Characterization of the NIR-LED was carried out in a nitrogen-filled glovebox. Current density-voltage (J-V) characteristics were recorded by Keithley 2400 source meter. Forward-viewing spectral radiant flux was measured by an integrating sphere coupled with a QE65 Pro spectrometer. The active device area was 0.035 cm^2.

Film characterizations. The PL and absorption images are taken with an upright fluorescence microscope (Nikon LV-100ND) integrated with a hyperspectral camera (Nireos Hera). A fiber-coupled femtosecond laser (Light conversion Pharos/Hero) is used as the excitation source for the

PL maps. Absorption images are taken with the microscope's transmission mode by coupling a Xenon lamp to the condenser. PL and absorption maps are measured with the same spot, ensuring perfect spatial overlapping. The synchrotron radiation-based GIWAXS measurements were performed at BL202 beamline, Shanghai Synchrotron Radiation Facility (SSRF). The diffraction patterns were collected by two-dimensional MarCCD 225 detector with a distance of 467 mm from samples to the detector. The wavelength of the X-ray was 1.24 Å (10 KeV). All samples were protected by a N_2-refilled box during measurements. Scanning electron microscopy (SEM, Zeiss) was operated at 5 keV to characterize the morphology of the samples and at 10 keV for EDS. The ATR-FT-IR spectra (2 cm^{-1} resolution) were recorded from a PIKE MIRacle ATR accessory with a diamond prim in a Vertex 70 Spectrometer (Bruker) using a DLaTGS detector at room temperature. The excitation density dependent PLQY measurement was pumped by a 515 nm pulse laser and collected with a Maya 2000 Pro visible spectrometer. For TA characterization an amplified femtosecond laser (Light Conversion Pharos) generated pulses of ~280 fs centred at 1030 nm with a repetition rate of 1 kHz. A broadband white light probe is generated by focusing the pulses into a thin sapphire plate. At short delays (<5 ns), the second harmonic of the fundamental provided the pump light (515 nm). At long delays (>1 ns), pump light at 532 nm was provided by the second harmonic of a Q-switched Nd:Yag laser (Innolas Picolo), which was electronically triggered and synchronized to the femtosecond laser via an electronic delay. For TRPL, an Andor spectrometer paired with an iStar iCCD is used for the detection. The camera acquisition is synchronized with the 1 kHz trigger signal from the laser (Light Conversion Pharos). The sample is excited with the second harmonic of the fundamental (515 nm) while the photoemission dynamics are recorded by gating the camera’s acquisition over a temporal window of 2 ns collected at increasing delay from the trigger reference (2 ns steps). The overall time resolution of the system is 2 – 4 ns. Kinetics are obtained by integrating the whole PL spectrum recorded at each delay.

The carrier densities were estimated using optical density and pump fluence as the equation S1

$$n = \frac{P \cdot \lambda_{ex} \cdot I_A(\lambda_\gamma) \cdot 2.303}{RR \cdot hc \cdot A_P \cdot L} \qquad \text{S1}$$

where P is the incident power, λ_{ex} is the wavelength of laser, RR is the laser repetition rate, A_P is the spot area on the sample, $I_A(\lambda_{ex})$ is the absorbance at the wavelength of the laser, and L is the film thickness in cm.

First-principles calculations. Quantum espresso software packages has been utilized to simulate all calculations in the supercell approach. The Perdew–Burke–Ernzerhof (PBE) exchange correlation functional was employed, together with ultrasoft pseudopotentials (shells explicitly included in calculations: I 5s, 5p; N, C 2s, 2p; O 2s 2p; H 1s; Sn 6s, 5p, 4d) with a cutoff on the wavefunctions of 40 Ry and a cutoff on the charge density of 320 Ry the DFT–D3 Van der Waals corrections. We have performed calculations at the Brillouin zone (BZ) Γ point. By maintaining the cell parameters at the experimental levels, DDS adsorption was simulated in the 2×2 in-plane (001) termination slab model of $FASnI_3$, which was constructed from the tetragonal phase of $FASnI_3$. A non-periodic vacuum of 15 Å has been applied in each system, perpendicular to the slabs. Finally, the adsorption energies were calculated with the following equation:

$$E_{\mathrm{Ads}} = E_{\mathrm{Adsorbed}} - E_{\mathrm{Decoupled}}.$$

Here, E_{Adsorbed} is the energy of the adsorption configuration, and $E_{\mathrm{Decoupled}}$ is the energy of the decoupled system, including the clean system and the single molecule.

Cyclic Voltammetry Oxidation half-potential ($E_{\mathrm{p/2}}$) of 4,4’-diaminobiphenyl sulfone (DDS) was obtained with a standard set of conditions to maintain internal consistency. Cyclic voltammograms were collected with Biologic VSP Potentiostat. Samples were prepared with 0.06 mmol of DDS in 6 mL of 0.1 M tetra-n-butylammonium tetrafluoroborate (TBATFB) in degassed acetonitrile. Measurements employed a three-electrode system, with a glassy carbon working electrode, a platinum wire counter electrode, a 0.1 M TBATFB $Ag/AgNO_3$ reference electrode, and a scan rate of 100 mV s^{-1}. Oxidative potentials have been referred to as the internal standard ferrocene (Fc). The absolute potential of SHE is 4.44eV. The HOMO of DDS is obtained from the following calculations:

$E_{\mathrm{vs.SHE}}$= 0.89V+0.29V ($Ag/AgNO_3$ as referring oxidative potentials) =+1.18 vs. SHE

$E_{\mathrm{vs.SHE}}$= 0.77V+0.40V (Fc^+/Fc as referring oxidative potentials) =+1.17 vs. SHE

The almost same values (+1.18 V and +1.17V) indicate the responsibility of our results.

Hence, the $\mathrm{HOMO(eV)}_{\mathrm{DDS}} \approx -(1.17+4.44) = -5.61$eV

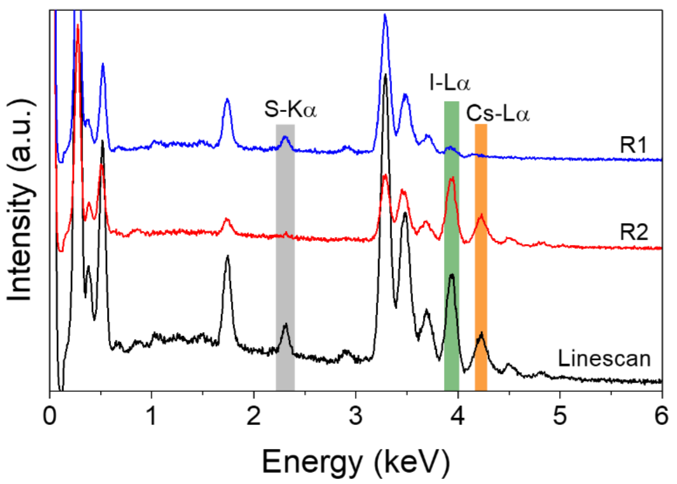

Figure S1. a EDS spectra from selected range of the stable tin-iodide perovskite thin film with DDS.

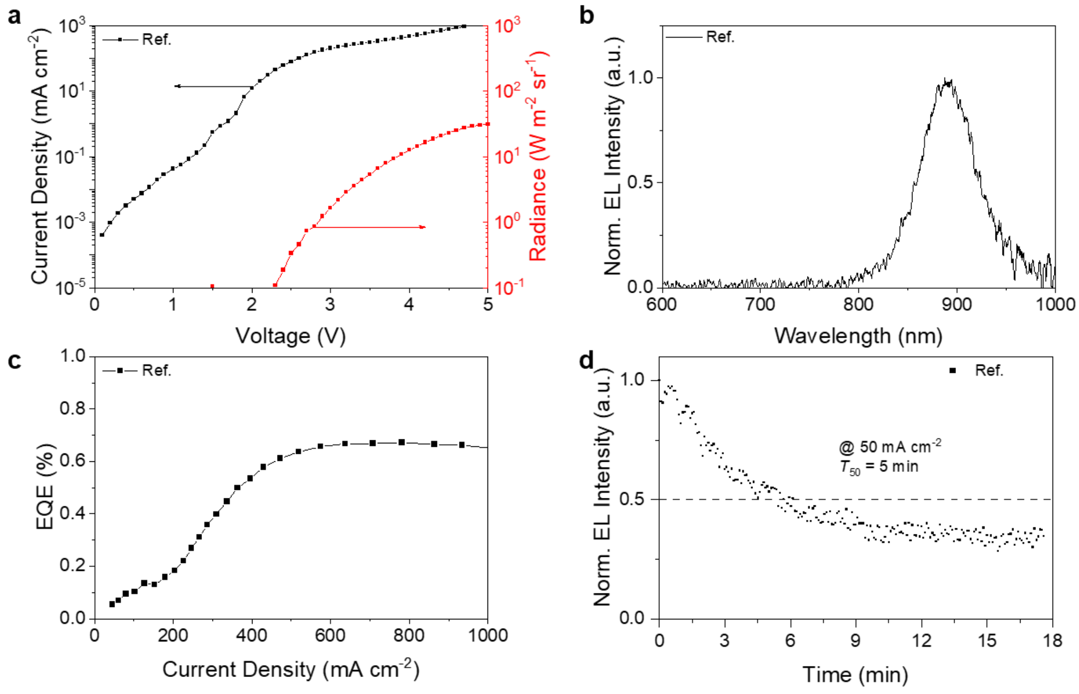

Figure S2. a Current density-voltage-radiance curves, **b** normalized EL spectrum at 4.0 V voltage, **c** EQE-current density curve, and **d** operational stability of the NIR-LEDs based on tin-iodide perovskite film without DDS.

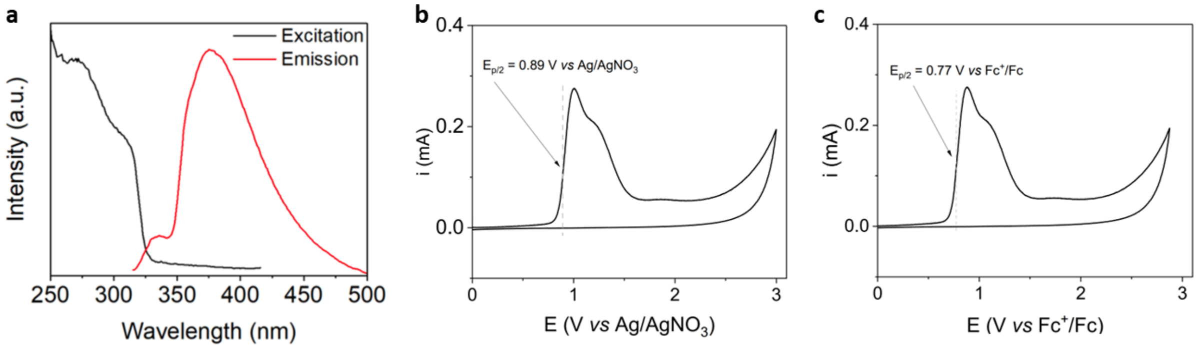

Figure S3. a Excitation and emission spectra of DDS film at room temperature. Cyclic Voltammetry of DDS, **b** obtaining $E_{p/2}$ = 0.89 V vs $Ag/AgNO_3$ and **c** obtaining $E_{p/2}$ = 0.77 V vs Fc^+ /Fc.

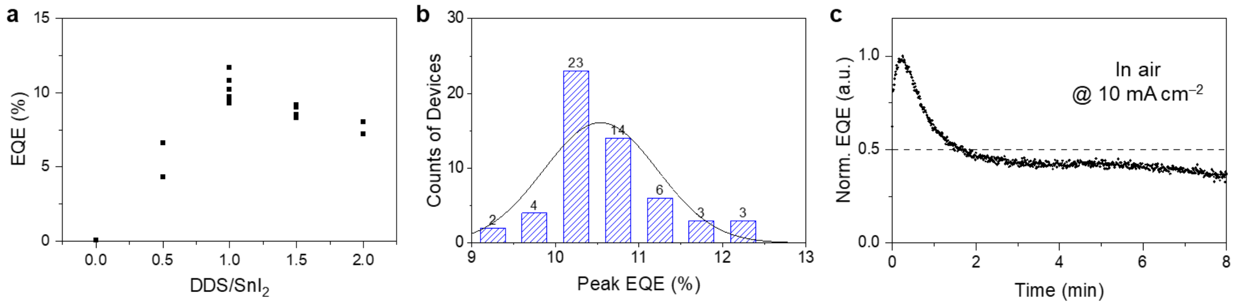

Figure S4. a Peak EQEs of NIR-LEDs based on tin-iodide perovskite films with various amount of DDS and **b** the peak EQEs histogram of the NIR-LEDs based on 1.0 DDS, which are measured in a N_2-filled glovebox. **c** The operational lifetime of optimized NIR-LEDs based on tin-iodide perovskite film measured in air.

Table S1. Summary of the representative device performance of NIR-LEDs based on tin-halide perovskites.

Perovskites	Peak EQE (%)	EL peak (nm)	Half-lifetime (h)	Ref.
$CsSnI_3$	3.8	950	--	Adv Mater. 2016, 28(36), 8029-8036
$MASn(Br_{1-x}I_x)_3$	0.72	667/945	--	J Phys Chem Lett. 2016, 7(14), 2653-2658
$CsSnI_3$+PEAI	3	920	2	J Phys Chem Lett. 2019, 10(3), 453-459
$CsSnI_3$	5.4	945	23.6	Adv Mater. 2021, 33(44), e2104414
$FASnI_3$	5.3	866	--	Adv Mater. 2022, 34(37), e2203180
$CsSnI_3$	5.3	965	--	ACS Energy Lett. 2023, 8(3), 1597-1605
$FACsSnI_3$	8.3	894	3	Nat. Photonics. 2023, 17(9), 755-760
$CsSnI_3$	2.63	948	39.5	Nat. Photonics. 2024, 18(2), 170-176
$FACsSnI_3$	11.6	898	0.38	Nat. Nanotechnol. 2024, 19(5), 632-637
$CsSnI_3$	6.6	935	17	Adv. Funct. Mater. 2023, 34(13), 2310530
$CsSnI_3$	5.6	931	24.58	InfoMat. 2024, 6(5), e12537
**$FACsSnI_3$**	**12.4**	**909**	**1.1**	**This work**

Table S2. The adsorption energy difference (E_{Ads}) between DDS and tin-iodide perovskites.

	E_{Ads} (eV) with DDS	E_{Ads} (eV) without DDS
DDS@FASnI_3-FAI-Terminal [-NH_2]	-1.99	-0.23
**DDS@FASnI_3-FAI-Terminal [-O]**	-2.65	-1.21
DDS@FASnI_3-SnI_2-Terminal [-NH_2]	**-3.12**	**-1.81**
**DDS@FASnI_3-SnI_2-Terminal [-O]**	-2.33	-0.69

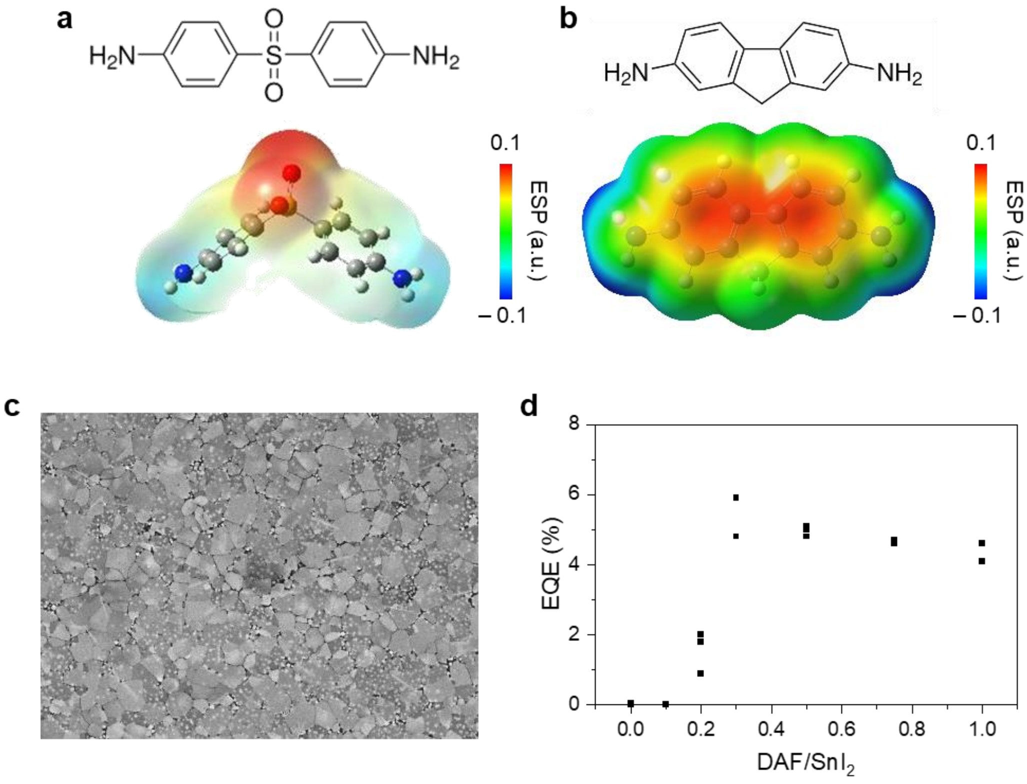

Figure S5. Chemical structure and simulated electrostatic potential of **a** DDS and **b** 2,7-Diaminofluorene (DAF). **c** Top-view SEM image of the tin-iodide perovskite thin film with DAF. **d** The EQEs of NIR-LEDs based on tin-iodide perovskite thin films with various amount of DAF.

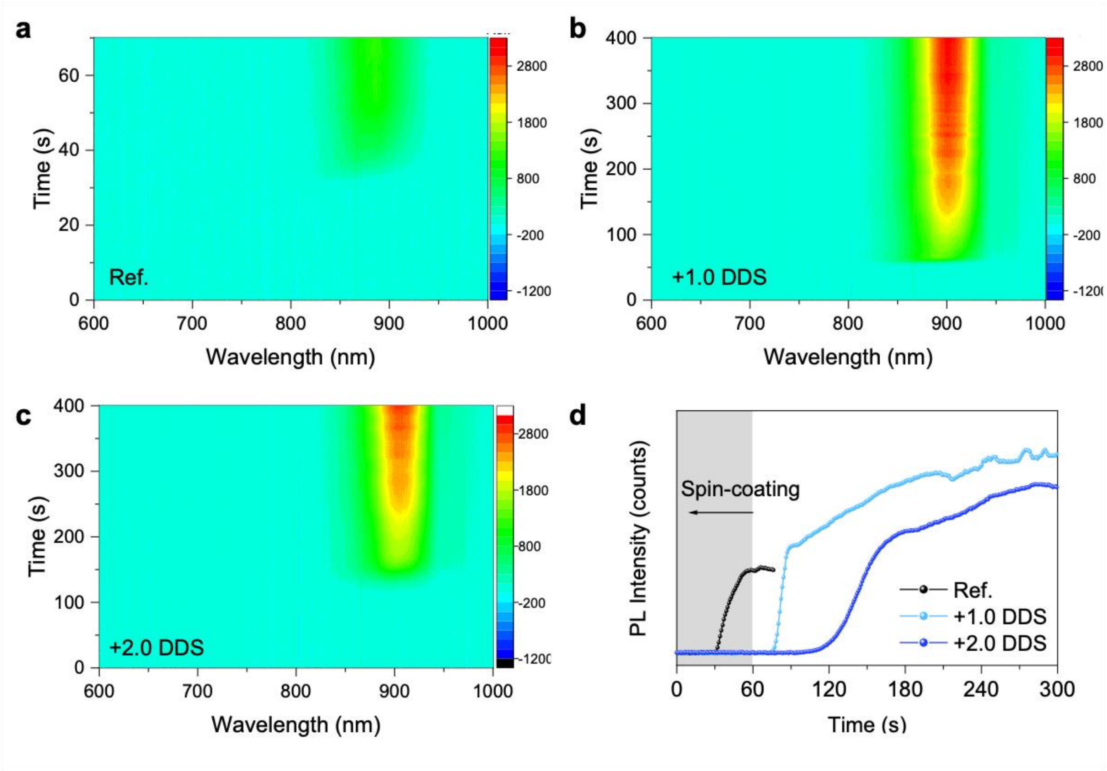

Figure S6. a – c PL spectra and **d** the integrated PL intensity of tin-iodide perovskite films during and after the spin-coating process.

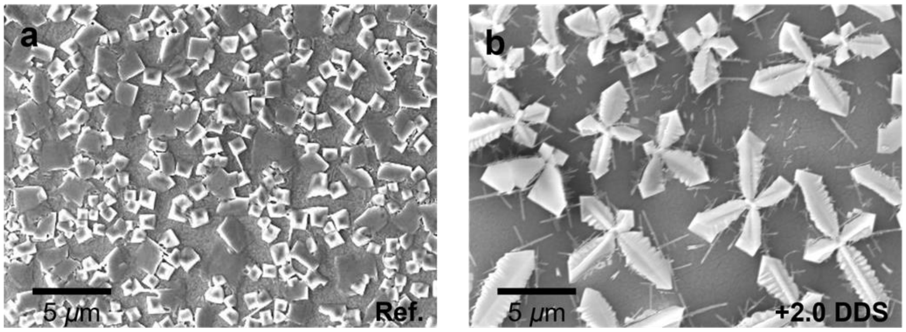

Figure S7. Top-view SEM images of the tin-iodide perovskite thin film with different amount of DDS.

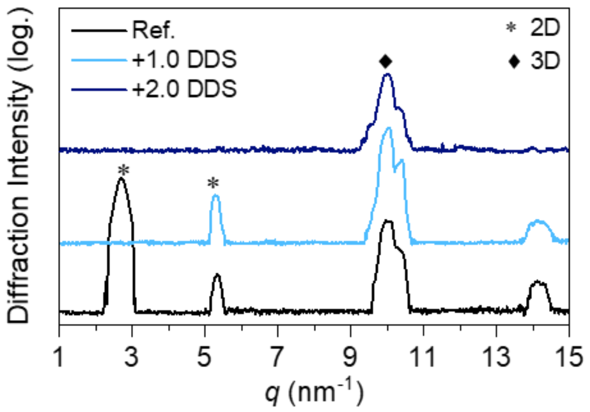

Figure S8. The integrated intensity of tin-iodide perovskite films without (Ref.) and with varying amounts of DDS.

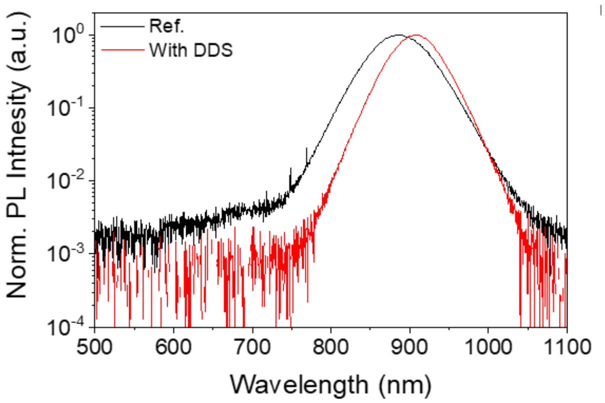

Figure S9. PL spectra of the tin-iodide perovskite films without (Ref.) and with DDS are excited by a 515 nm pulse laser.

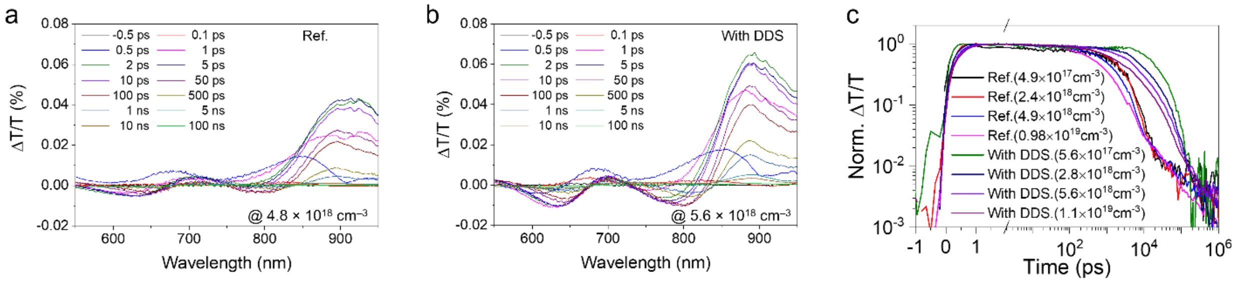

Figure S10. TA spectra of the tin-iodide perovskite films **a** without (Ref.) and **b** with DDS. **c** TA decays of the main photobleach at various excitation densities.

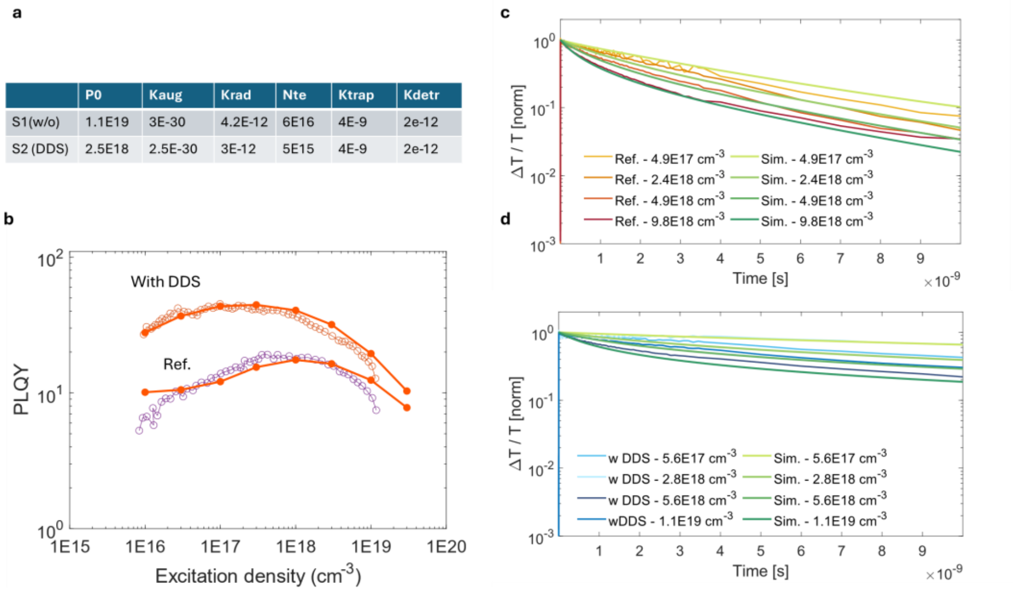

	P0	Kaug	Krad	Nte	Ktrap	Kdetr
S1(w/o)	1.1E19	3E-30	4.2E-12	6E16	4E-9	2e-12
S2 (DDS)	2.5E18	2.5E-30	3E-12	5E15	4E-9	2e-12

Figure S11. a Parameters (doping, Auger rate, radiative rate, trap density, trapping rate, rate of recombination of a trapped electron with free hole) adopted to simulate **b** PLQY and TA photobleach decays for different excitation density in case of the tin-iodide perovskite films **c** without (Ref.) and **d** with DDS.
